# Interactions between the FTO and GNB3 Genes Contribute to Varied Clinical Phenotypes in Hypertension

**DOI:** 10.1371/journal.pone.0063934

**Published:** 2013-05-14

**Authors:** Rahul Kumar, Samantha Kohli, Perwez Alam, Ritankur Barkotoky, Mohit Gupta, Sanjay Tyagi, S. K. Jain, M. A. Qadar Pasha

**Affiliations:** 1 Functional Genomics Unit, Council of Scientific and Industrial Research -Institute of Genomics and Integrative Biology, Delhi, India; 2 Department of Cardiology, G. B. Pant hospital, New Delhi, India; 3 Department of Biotechnology, Hamdard University, New Delhi, India; University of Bonn, Institut of Experimental Hematology and Transfusion Medicine, Germany

## Abstract

**Background:**

The genes *FTO* and *GNB3* are implicated in essential hypertension but their interaction remains to be explored. This study investigates the role of interaction between the two genes in the pathophysiology of essential hypertension.

**Methods/Principal Findings:**

In a case-control study comprising 750 controls and 550 patients, interaction between the polymorphisms of *FTO* and *GNB3* was examined using multifactor dimensionality reduction (MDR). The influence of interaction on clinical phenotypes like systolic and diastolic blood pressure, mean arterial pressure and body mass index was also investigated. The 3-locus MDR model comprising *FTO* rs8050136C/A and *GNB3* rs1129649T/C and rs5443C/T emerged as the best disease conferring model. Moreover, the interacted-genotypes having either 1, 2, 3, 4 or 5 risk alleles correlated with linearly increasing odds ratios of 1.91 (*P* = 0.027); 3.93 (*P* = 2.08E–06); 4.51 (*P* = 7.63E–07); 7.44 (*P* = 3.66E–08) and 11.57 (*P* = 1.18E–05), respectively, when compared with interacted-genotypes devoid of risk alleles. Furthermore, interactions among haplotypes of *FTO* (H_1_−_9_) and *GNB3* (H_a-d_) differed by >1.5-fold for protective-haplotypes, CTGGC+TC [H_2_+H_a_] and CTGAC+TC [H_4_+H_a_] (OR = 0.39, *P* = 0.003; OR = 0.22, *P* = 6.86E–05, respectively) and risk-haplotypes, AAAGC+CT [H_3_+H_c_] and AAAGC+TT [H_3_+H_d_] (OR = 2.91, *P* = 9.98E–06; OR = 2.50, *P* = 0.004, respectively) compared to individual haplotypes. Moreover, the effectiveness of gene-gene interaction was further corroborated with a 1.29-, 1.25- and 1.38-fold higher SBP, MAP and BMI, respectively, in patients having risk interacted-haplotype H_3_+H_c_ and 2.48-fold higher SBP having risk interacted-haplotype H_3_+H_d_ compared to individual haplotypes.

**Conclusion:**

Interactions between genetic variants of *FTO* and *GNB3* influence clinical parameters to augment hypertension.

## Introduction

Essential hypertension (EH) is a risk predictor of stroke and cardiovascular diseases and results in high mortality [1]. Studies in the last few decades have established the significance of various physiological pathways in EH [2], including the importance of the relative interactions between the autonomic nervous system (ANS) and G protein-coupled receptors (GPCRs) in the regulation of blood pressure (BP) [3]–[6]. Subsequent ongoing cohort studies have revealed that 40–60% of BP variability is genetically determined [7]–[9]. Among the various genes of these pathways, fat mass and obesity associated (*FTO*) and guanine nucleotide binding protein, β-polypeptide 3 (*GNB3*) appear relevant because the former is highly expressed in BP regulating centers of hypothalamus and the latter is involved in intracellular signaling pathways. Recent genome wide and meta-analysis reports have associated both individual genes with hypertension promoting risk factors e.g., BMI and adiposity especially in Asians [10]–[15].

*FTO*, originally identified in mice with fused toes, is highly expressed in paraventricular and dorsomedial nuclei of the hypothalamus [16]. Genome-wide linkage studies have identified linkages between *FTO* and BP [17], [18]. Similarly, *GNB3,* encoding the Gβ3 subunit of heterotrimeric signal transducing G proteins [19] has polymorphisms that have shown to be associated with susceptibility to EH [20]–[22].

Interestingly, the interactive role of *FTO* and *GNB3* has not been studied despite the known role of both the genes in BP regulation. As EH is a multifactorial disease, the interaction between these two genes may be crucial. To address this question, we screened the potential single nucleotide polymorphisms (SNPs) of *FTO* and *GNB3* in a case-control design and looked for their interactive effect in hypertension pathohysiology in correlation with clinical parameters including systolic blood pressure (SBP), diastolic blood pressure (DBP), mean arterial pressure (MAP) and body mass index (BMI).

## Materials and Methods

### Ethics Statement

The study protocol and consent form were approved by human ethics committee of both CSIR-IGIB and GB Pant hospital. Prior to written consent, subjects were informed of the objectives, study organization and implications of their participation.

### Study Participants

Ethnically-matched consecutive unrelated 4000 North-Indian participants, over a period of 4 years, were screened in the hypertension and general outpatient clinic of GB Pant hospital, New Delhi. A significant number of subjects were excluded because they did not give consent for the study, were on medication, and to maintain both the age limit and the male to female ratio in the two groups. Moreover, physical examination and laboratory tests excluded individuals with coronary artery disease, vascular disease, stroke, secondary hypertension, diabetes mellitus and renal diseases. In the final analysis we included 1300 case-control participants comprising of 750 controls and 550 patients.

### Inclusion Criteria and Clinical Evaluation

Controls recruitment criteria included: age 25–60 years, SBP<120 mmHg and DBP<80 mmHg, absence of family history of hypertension and any disease medication. Patients recruitment criteria was: age 25–60 years, SBP≥140 mmHg and/or DBP≥90 mmHg (JNC VII) and absence of antihypertensive medication. All the subjects were rested for 5 minutes prior to BP measurement. Three measurements of BP, in supine position, using a calibrated mercury sphygmomanometer with appropriate adult cuff size were recorded by the clinicians. The point at which the first of two or more Korotkoff sound was heard was recorded as SBP and the disappearance of Korotkoff sound as DBP. Blood was drawn in supine position after overnight fasting. Peripheral blood leucocytes were used for DNA extraction and plasma for the analysis of biochemical parameters; samples were stored at −40°C if not used immediately.

### Routine Biochemical Assays

Total cholesterol, triglycerides, glucose and uric acid were estimated on a high-throughput autoanalyzer (Elecsys 2010, Roche, Germany) and SpectraMax384 spectrophotometer (Molecular Devices, Sunnyvale CA, USA). All the measurements were performed in duplicate. The intra- and inter-assay coefficient of variations were <5% for all the measurements.

### Selection of FTO and GNB3 SNPs

Selection of SNPs was based on their location in respective genes, clinical and functional relevance, and their association with hypertension [13], [18], [21], [22]. Selection was also based on their tagging with other SNPs (www.hapmap.org) and association with BMI, obesity and diabetes that affect BP [10], [12], [23]–[26]. Among the *FTO* SNPs, rs8050136C/A, rs9939609T/A, rs9926289G/A, rs9930506A/G, rs9932754T/C, rs9933040A/T and rs62033414C/G were from intron 1; rs16952624C/T (Ala405Val) was from exon 9 and rs16953075C/T was from the 3' UTR. In case of *GNB3* SNPs, rs1129649T/C (Ile685Thr) was from exon 1 and rs5443 (825C/T) was from exon 10. The selected SNPs cover around 16 kb and 4 kb of the *FTO* and *GNB3* genes, respectively.

### Genotyping

Genomic DNA was isolated from peripheral blood leukocytes using a standard protocol. All the nine SNPs of *FTO* and rs5443C/T SNP of *GNB3* were analyzed by SNaPshot ddNTP primer extension PCR (Applied Biosystems, Foster City, USA). The *GNB3* rs1129649T/C was genotyped by PCR-restriction fragment length polymorphism (RFLP). Two observers independently read and confirmed all the genotypes; discrepancies, if any, were resolved by repeating PCR-RFLP and SNaPshot. The primers, optimal conditions for amplification and restriction enzymes for digestion are presented in Tables S1 and S2.

### Haplotypes and Linkage Disequilibrium

Haplotypes were estimated from genotypes using software PHASEv2.1.1 [27] and the best haplotypes were identified for protection and risk. Order of SNPs in inferred-haplotypes was based on their physical location, starting from SNPs at the upstream promoter region to downstream. Distribution of each haplotype was compared using multivariate logistic regression analysis. Haplotypes with <2% frequency were excluded. The extent of association, i.e., the Lewontin’s coefficient (D′) and squared correlation coefficient (r^2^) for pairwise linkage disequilibrium (LD), was calculated by Haploview-v4.0 [28].

### Gene-Gene Interactions

Gene-gene interactions (epistasis), in same subjects, were analyzed in two ways, using (1) interacted-genotypes and (2) interacted-haplotypes.

#### (1) Interacted-genotypes

The interacted-genotypes between *FTO* and *GNB3* were analyzed using multifactor dimensionality reduction (MDR-v.1.2.2) software [29]. The best disease predicting MDR model was identified on the basis of interacted-genotypes carrying different set of risk alleles using the gene counting method. The *P* value and odds ratio (OR) were calculated using multivariate logistic regression analysis after adjustment with seven confounders namely, age, gender, BMI, alcohol, smoking habit, triglyceride, cholesterol and also by Bonferroni’s correction test for multiple testing.

#### (2) Interacted-haplotypes

In this analysis, we first inferred risk and protective haplotypes of each gene on the basis of *P* value and OR at 95% confidence interval (CI). We then looked for haplotype-haplotype interactions through the interaction of risk or protective haplotypes between *FTO* and *GNB3* using Haploview-v4.0 [28], Hap Evolution [30] and the gene counting method. Statistical significance was determined empirically using multivariate logistic-regression model after adjustment with seven confounders (the same as used for interacted-genotypes above) and Bonferroni’s correction test for multiple testing.

### Correlation Analysis

To strengthen the genetic outcome, the investigated SNPs were analyzed for possible correlation with clinical characteristics. Genotypes and haplotypes were correlated with SBP, DBP, MAP and BMI. Likewise, both the interacted-genotypes and interacted-haplotypes of *FTO* and *GNB3* were correlated with the same clinical parameters to determine the extent of gene-gene interaction.

### Statistical Analysis

Unpaired Student’s *t*-test (two-tailed) was performed to compare the differences in baseline clinical and demographic characteristics between the two groups. A goodness-of-fit test was used for testing the Hardy-Weinberg Equilibrium (HWE) using DeFinetti program (http://ihg.gsf.de/cgi-bin/hw/hwa1.pl). Allele and genotype frequencies between the study subjects were estimated by χ^2^ test. The allelic distribution between our population and HapMap populations was compared after retrieving the data from www.hapmap.org. The risk of having hypertension was estimated as an odds ratio (OR) at 95% confidence interval (95% CI) using multivariate logistic regression analysis by SPSS-12 (SPSS Inc., Chicago, Illinois, USA). Haplotypes distribution was compared by multiple regression analysis based on the frequency of each haplotype individually versus all others combined between both the groups. The clinical parameters were expressed as mean ± SD. Further, *P* value and estimated difference at 95% CI for continuous variables e.g., SBP, DBP, MAP and BMI against categorical variables e.g., individual and interacted genotypes and haplotypes were analyzed using a general linear model (GLM) after adjustment for the seven confounding factors. The transcription factor binding site (TFBS) with respect to the studied SNPs was analyzed using *TFSEARCH* developed by Yutaka Akiyama *(* http://www.rwcp.or.jp/papia *)*. The power of association at α = 0.05 was calculated using EPIINFO ver.6. A *P* value of <0.05 was considered statistically significant.

## Results

### Comparison of Demographic and Clinical Characteristics

Patients had significantly higher BMI (*P* = 0.003), clinical parameters e.g. SBP, DBP and MAP (*P*<0.0001, each) and the levels of routine biochemical parameters e.g., cholesterol and triglyceride (*P*<0.0001, each) when compared with controls (Table 1).

**Table 1:** Demographic and clinical characteristics of studied participants.

Parameters	Patients	Controls	*P*
	n = 550	n = 750	
*Gender*			
Male	467(85%)	649(87%)	–
Female	83(15%)	101(13%)	–
*Clinical characteristics*			
Age, year	49.8±11.0	48.5±13.0	NS
BMI, kg/m^2^	25.0±3.7	24.0±7.4	0.003
SBP, mmHg	159.4±17.8	117.6±8.0	<0.0001
DBP, mmHg	96.4±9.0	77.6±3.9	<0.0001
MAP, mmHg	116.9±12.7	91.0±21.2	<0.0001
*Biochemical parameters*			
Total cholesterol, mmol/L	3.3±1.2	2.4±1.3	<0.0001
Triglycerides, mmol/L	1.3±0.8	1.0±0.6	<0.0001
Uric acid, mg/dl	4.7±1.6	4.6±1.4	NS
Glucose, mg/dl	101.0±22.0	98.1±32.0	NS
Protein urea	Nil	Nil	–
*Life style/history*			
Diet, non-veg	68%	30%	–
Family history, EH	78%	None	–
Alcohol	15%	10%	–
Smoking history	25%	15%	–

Data are presented as mean ± standard deviation; n, number of subjects; BMI, body mass index; SBP, systolic blood pressure; DBP, diastolic blood pressure; MAP, mean arterial pressure; EH, essential hypertension. *P*-values were calculated using EPIINFO ver.6 (Center for Disease Control, Atlanta, Georgia, USA) software.

### Single-locus Association Analyses

The allele and genotype frequencies of studied SNPs were in HWE **(** *P*>0.05, Table S3). The allele frequency of the studied SNPs was comparable with HapMap Caucasian population (*P*>0.05, Table S4). The single-locus genotype distribution is shown in Table S5. The *FTO* alleles rs8050136A (*P* = 0.014), rs9939609A (*P* = 0.002), rs9926289A (*P* = 0.015) and the *GNB3* alleles rs1129649C (*P* = 8.76E–06) and rs5443T (*P* = 9.45E–10) were associated with increased risk of hypertension.

### Identification of Risk or Protection Associated Haplotypes

The pairwise LD was similar for both the groups (Figure S1). At >2% cutoff frequency, 9 haplotypes for *FTO* and 4 haplotypes for *GNB3* were inferred (Figure S2). For convenience, the *FTO* haplotypes were marked as H_1–9_ and the *GNB3* haplotypes as H_a-d_. The haplotypes *FTO* H_3_: AAAGC and *GNB3* H_c_: CT and H_d_: TT increased the risk of hypertension with ORs of 1.48 (*P* = 0.005), 1.74 (*P* = 4.36E–05) and 1.79 (*P* = 1.79E–04), respectively, while haplotypes *FTO* H_4_: CTGAC and *GNB3* H_a_: TC with ORs of 0.38 (*P* = 5.45E–05) and 0.49 (*P* = 2.12E–11), respectively, were protective. The omnibus global test for both *FTO* and *GNB3* haplotypes showed significant association with hypertension (*P*<0.0001, each).

### Gene-gene Interaction and Hypertension Risk

#### (A) Interacted-genotypes

**Figure 1:**
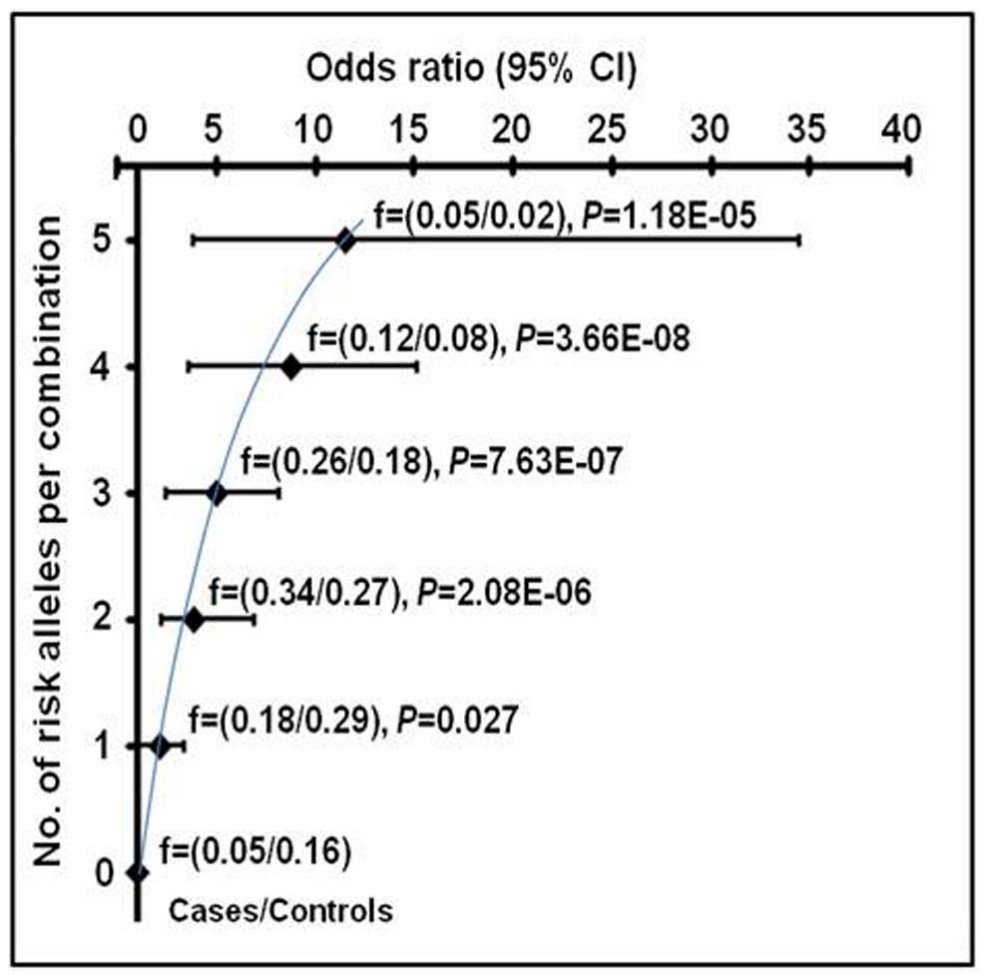
Interacted-genotypes carrying varied number of risk alleles of the 3-locus best model in hypertension susceptibility. The MDR model consisted of the SNPs *FTO,* rs8050136C/A and *GNB3*, rs1129649T/C and rs5443C/T. Stratification was done on the basis of interacted-genotypes carrying risk alleles from 0 to 5. f, represents the frequency of the risk alleles in an interacted-genotypes. *P*-value and OR at 95% CI were calculated after adjustment for age, gender, BMI, alcohol, smoking, triglyceride and cholesterol using multivariate logistic regression analysis. The threshold *P* value was (0.05/6) = 0.008.

The exhaustive data mining MDR analysis was used to evaluate the impact of interactions among the genotypes of the eleven SNPs of *FTO* and *GNB3* on hypertension; Table 2 summarizes the results obtained for 2-locus to 7-locus models. The 3-locus model comprised of the polymorphisms *FTO* rs8050136C/A and *GNB3* rs1129649T/C and rs5443C/T emerged as the best disease predicting model with the highest level of statistical significance (TA = 0.62, CVC = 9/10; OR = 3.9, *P* = 0.0005) and a prediction error of 0.38. Out of the seven expected interacted-genotypes, only six interacted-genotypes were obtained, with the number of risk alleles varying between 0 and 5. Of note, the five interacted-genotypes bearing 1, 2, 3, 4 and 5 risk alleles corresponded with linearly increasing ORs, which varied between 1.91 and 11.57 (*P* = 0.027−3.66E–08, Figure 1).

**Table 2:** Interaction between genotypes of *FTO* and *GNB3* using MDR.

FTO+GNB3	Best models	TB	TA	CVC	P value	OR(95% CI)
2L	rs1129649T/C rs5443C/T	0.62	0.59	8/10	0.067	2.1(0.9–4.8)
3L†	rs8050136C/A rs1129649T/C rs5443C/T	0.63	0.62	9/10	0.0005	3.9(1.8–8.5)
4L	rs9930506A/G rs9932754C/T rs1129649T/C rs5443C/T	0.65	0.61	4/10	0.006	3.0(1.4–6.5)
5L	rs9939609T/A rs9930506A/G rs9932754C/T rs1129649T/C rs5443C/T	0.64	0.61	8/10	0.0002	4.9(2.0–11.7)
6L	rs8050136C/A rs9939609T/A rs9926289G/A rs9932754C/T rs1129649T/C rs5443C/T	0.63	0.59	6/10	0.0165	3.0(1.2–7.7)
7L	rs8050136C/A rs9939609T/A rs9926289G/A rs9930506A/G rs9932754C/Trs1129649T/C rs5443C/T	0.61	0.58	10/10	0.012	3.6(1.3–10.4)

† Overall best MDR model; TB, Testing balance accuracy; TA, Training accuracy; CVC, Cross validation consistency. 2L–7L, represents 2-locus to 7-locus MDR model carrying best interacted genotypes. *P* values were calculated by permuting the cases and controls 1000 times.

#### (B) Interacted-haplotypes

**Figure 2:**
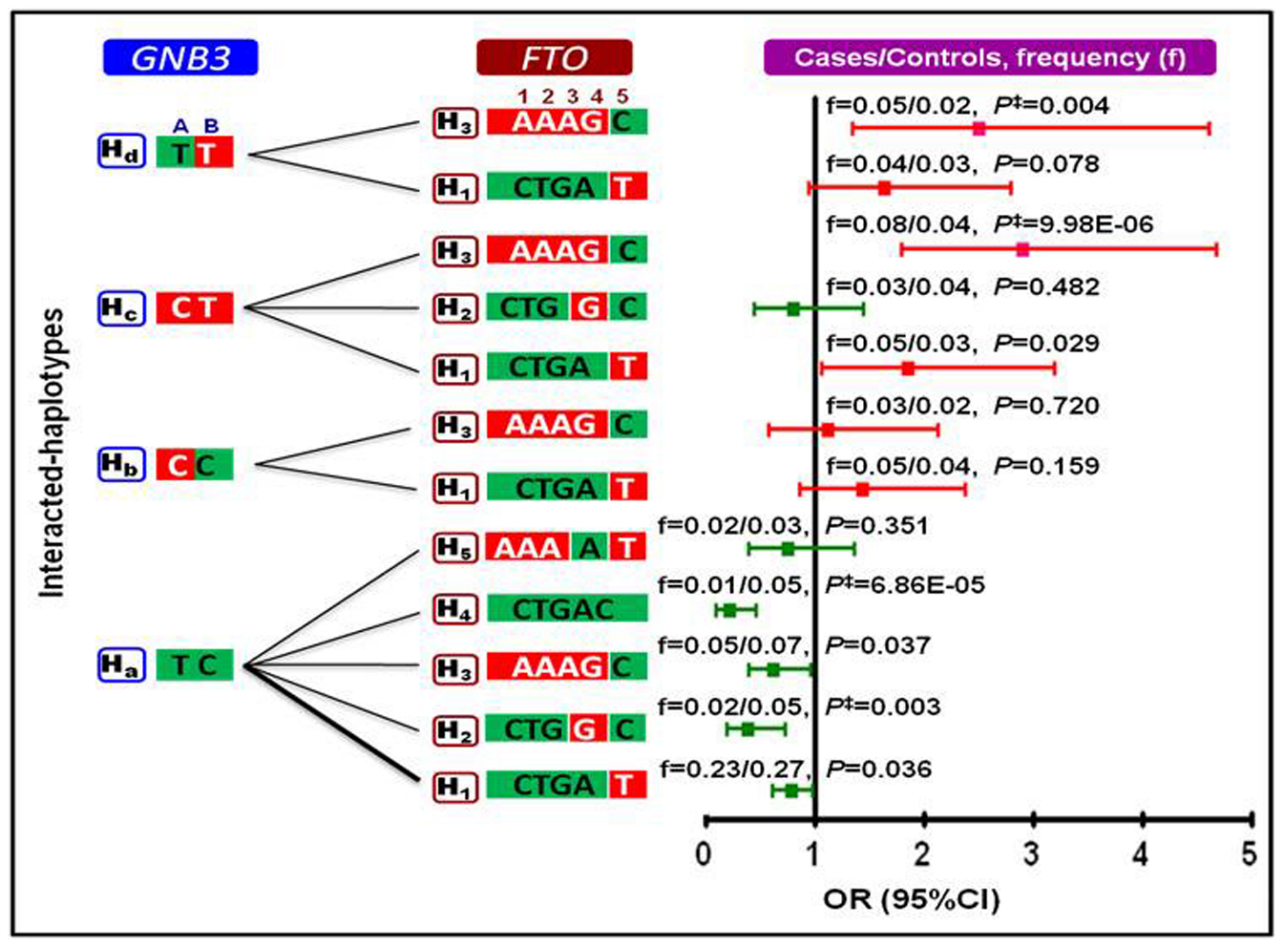
Interacted-haplotypes of *FTO* and *GNB3* in cases and controls. The red and green shaded areas in the haplotypes block represent the risk and non-risk alleles, respectively. The interactions were examined at a frequency of >2%. The thin and thick line represents the interaction frequency of >2% and 10%, respectively. H represents a haplotype, H with numeric and alphabet symbol represent the *FTO* and *GNB3* haplotypes, respectively. The alleles in each block belonged to SNPs A = rs1129649T/C and B = 825C/T of *GNB3* and 1 = rs8050136C/A, 2 = rs9939609T/A, 3 = rs9926289G/A, 4 = rs9930506A/G and 5 = rs9932754T/C of *FTO*. *P* ^‡^ value was statistically significant for risk and protective interacted-haplotypes. The *P*-value and OR were calculated after adjustment for potential confounding factors and Bonferroni’s correction. ‘f’, represents frequency of interacted-haplotypes. The threshold *P*-value was (0.05/7) = 0.007.

In this analysis, the 9 *FTO* and 4 GNB3 haplotypes were allowed to interact with each other and the haplotypes that showed significant interaction were selected. As shown in Figure 2, the *FTO* risk haplotype, H_3_: AAAGC interacted with *GNB3* risk haplotypes, H_c_: CT and H_d_: TT. The interacted-haplotypes H_3_+H_c_ and H_3_+H_d_ contributed to 1.8- and 1.5-fold increase in hypertension susceptibility than the individual risk haplotypes of either gene alone (*P* = 9.98E–06; *P* = 0.004, respectively). *FTO* protective haplotypes H_2_: CTGGC and H_4_: CTGAC interacted with *GNB3* protective haplotype H_a_: TC to contribute to 1.5- and 2.0-fold lower hypertension susceptibility than the individual protective haplotypes of either gene alone (*P* = 0.003; *P* = 6.86E–05, respectively). Furthermore, the risk alleles *FTO* rs8050136A and rs9932754T and *GNB3* rs5443T showed highest interaction ratio of 32%, 40% and 85%, respectively, and as a consequence all those haplotypes bearing these alleles associated with higher haplotype risk ratio (Figure S3).

### Correlation with Clinical Characteristics

#### (a) Genotypes/alleles versus clinical characteristics

**Figure 3:**
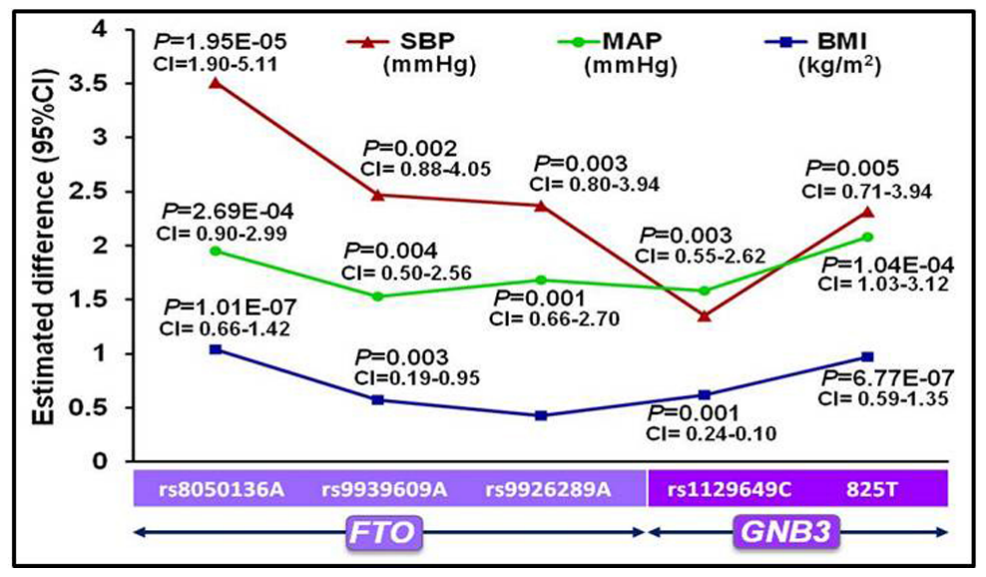
Difference in BP and BMI according to risk alleles of *FTO* and *GNB3* SNPs. CI represents confidence interval. The general linear model was used to calculate *P*-value and estimated difference at 95% CI after adjustment for potential confounding factors and Bonferroni’s correction.

The general linear model revealed a significant positive correlation of risk genotypes of *FTO* SNPs rs8050136C/A, rs9939609T/A and rs9926289G/A and *GNB3* SNPs rs1129649T/C and rs5443C/T with SBP, MAP and BMI (*P* = 0.005−3.96E–07). As a consequence, *FTO* risk alleles rs8050136A and rs9939609A correlated with 3.51 and 2.47 mmHg higher SBP (*P* = 1.95E–05; *P* = 0.002, respectively); 1.95 and 1.53 mmHg higher MAP (*P* = 2.69E–04; *P* = 0.004, respectively) and 1.04 and 0.57 kg/m^2^ higher BMI (*P* = 1.01E–07; *P* = 0.003, respectively). *FTO* risk allele rs9926289A correlated with 2.37 and 1.68 mmHg higher SBP and MAP (*P* = 0.003; *P* = 0.001, respectively; Figure 3). The *GNB3* risk allele rs1129649C correlated with 1.58 mmHg higher MAP (*P* = 0.003) and 0.62 kg/m^2^ higher BMI (*P* = 0.001); *GNB3* risk allele rs5443T correlated with 2.32 mmHg higher SBP (*P* = 0.005), 2.08 mmHg higher MAP (*P* = 1.04E–04) and 0.97 kg/m^2^ higher BMI (*P* = 6.77E–07).

#### (b) Haplotypes versus clinical characteristics

**Figure 4:**
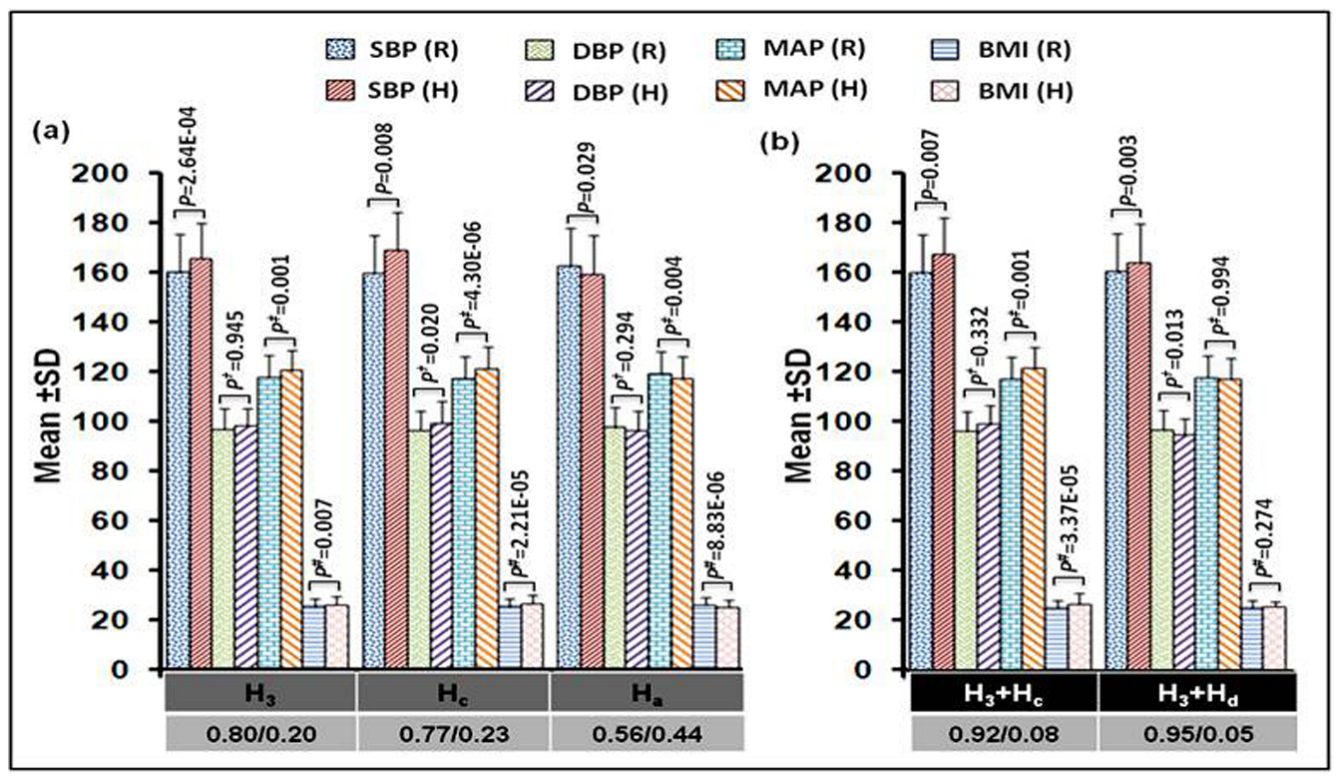
Correlation analyses for (a) individual haplotypes (b) interacted-haplotypes of *FTO* and *GNB3* with clinical parameters. The general linear model was used to calculate significance level after adjustment with age, gender, smoking, alcohol, triglyceride, cholesterol and Bonferroni’s correction test. Besides, *P* was adjusted for DBP, BMI; *P* ^†^ for SBP, BMI and *P* ^#^ for SBP, DBP. (R), represents reference for remaining haplotypes against any studied individual haplotype (H). The X-axis represents individual haplotypes H_3_: AAAGC, H_c_: CT and H_a_: TC and interacted-haplotypes H_3_+H_c_: AAAGC+CT and H_3_+H_d_: AAAGC+TT. The numerator and denominator represent frequency of (R) and (H), respectively.

As shown in Figure 4a, *FTO* risk haplotype H_3_ correlated with an increase of 3.59 mmHg SBP and 2.19 mmHg MAP(*P* = 0.002; *P* = 0.008, respectively). With respect to *GNB3* risk haplotype H_c,_ the increase was 2.53 mmHg SBP (*P* = 0.04), 2.85 mmHg MAP (*P* = 2.15E–05) and 0.97 kg/m^2^ BMI (*P* = 1.10E–04). The protective haplotype H_a_ correlated with a decrease of 1.52 mmHg MAP (*P* = 0.02) and 0.85 kg/m^2^ BMI (*P* = 4.41E–05).

#### (c) Interacted-genotypes versus clinical characteristics

**Figure 5:**
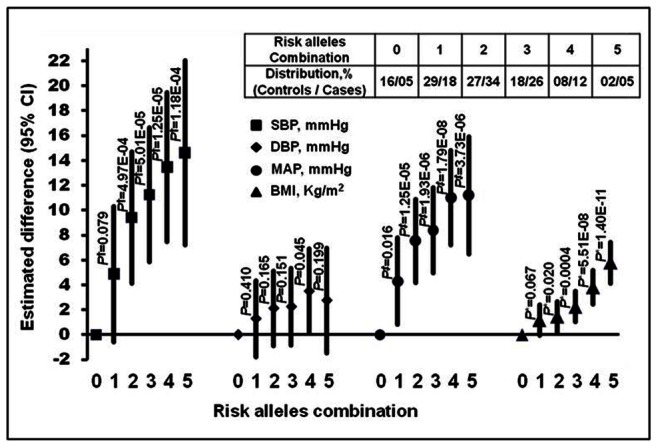
Variation in clinical characteristics according to number of risk-alleles in interacted-genotypes in 3-locus MDR model. The general linear model was used to calculate significance level after adjustment with age, gender, smoking, alcohol, triglyceride, cholesterol and Bonferroni’s correction test. Besides, *P* ^†^ was adjustment with BMI and DBP; *P* ^‡^ with BMI; P* with SBP, DBP. The threshold *P*-value was (0.05/10) = 0.005.

As shown in Figure 5, the interacted-genotypes bearing 1, 2, 3, 4 and 5 risk alleles correlated with linear increase in SBP of 4.88–14.62 mmHg (*P* = 0.079−1.18E–04), MAP 4.29–11.29 mmHg (*P* = 0.016−3.73E–06) and 1.16–5.78 kg/m^2^ BMI (*P* = 0.067−1.40E–11) when compared against interacted-genotypes without risk alleles.

#### (d) Interacted-haplotypes versus clinical characteristics

As shown in Figure 4b, an estimated increase of 3.96 mmHg SBP, 3.14 mmHg MAP and 1.11 kg/m^2^ BMI was observed in the presence of interacted-haplotype H_3_+H_c_ when compared against the remaining interacted-haplotypes from both the genes (*P* = 0.007; *P* = 0.001 and *P* = 3.37E–05, respectively). Of consequence, epistasis influence resulted in a 1.29-, 1.25- and 1.38-fold higher SBP, MAP and BMI, respectively, in the patients with interacted-haplotypes H_3_+H_c_ compared to individual risk haplotypes H_3_ and H_c_. The second risk interacted-haplotype, H_3_+H_d_ significantly correlated with an estimated increase of 5.44 mmHg SBP (*P* = 0.003), with epistasis contributing a 2.48-fold higher SBP.

### The SNPs and the Associated Transcription Factor

The transcription factor binding site (TFBS) in the presence of protective and risk alleles of both the genes changes the preference for the transcription factors (Figure S4). For example in the presence of rs8050136C allele, the transcription factors (TFs) were *CDP-CR* and *cap*; whereas in the presence of risk allele rs8050136A, the TFs were *CdxA*, *Abd-B* and *Croc*. Further, in the presence of *FTO* protective allele rs9930506A, the TFs were *Dfd* and *MATalp* and in the presence of risk allele rs9930506G, a single TF *Dfd* was noted. In the presence of *FTO* protective allele rs9932754T four TFs *HNF-3b*, *Cap*, *Skn-1* and *CdxA* were observed; whereas, in the presence of risk allele rs9932754C a single TF *HSF2* was observed. Likewise, in case of *GNB3*, the protective allele rs1129649T associated with three TFs *NIT2*, *Cap* and *NF-1* whereas, in the presence of risk allele rs1129649C, only TF *Cap* was associated.

## Discussion

In this study, the epistasis models of interacted-genotypes and interacted-haplotypes demonstrated increased hypertension susceptibility. Notably, stratification of the interacted-genotypes, as obtained in the best locus MDR model on the basis of presence of number of risk allele(s) in increasing order, correlated linearly with hypertension susceptibility. Furthermore, the interaction between *FTO* and *GNB3* when analyzed through haplotype-haplotype interactions revealed substantial modifications in the ORs for risk and protection compared to individual haplotypes. Moreover, the general linear model showed substantial correlation of interacted-genotypes and interacted-haplotypes with clinical characteristics, e.g., SBP, DBP, MAP and BMI.

Our findings on individual genes were significant as it revealed higher OR for EH in the presence of risk alleles of *FTO* rs8050136C/A, rs9939609T/A and rs9926289G/A, and *GNB3* rs1129649T/C and rs5443C/T SNPs, even after adjustment for potential confounders. Literature suggested an association of the *FTO* variants with hypertension [18], [23] or mediation through other hypertension risk factors like BMI or adiposity [10], [12], [31], [32]. Likewise, given the role of heterotrimeric G-proteins in intracellular signaling pathways, the *GNB3* variants were studied in EH [20]–[22], [33]. The rs5443C/T of *GNB3* was associated with enhanced activation of G protein-mediated signaling [20], noradrenaline-induced vasoconstriction [34], higher plasma sodium and lower potassium levels [33] and the C allele of rs1129649T/C was associated with salt sensitive BP [35].

Although our single locus results on *FTO* and *GNB3* were encouraging, however, in a polygenic and multifactorial disease like hypertension, the magnitude of effect is bound to be missed if the genes are examined individually and without considering potential interactions [36]. The evaluation of gene-gene interactions not only increases the power to detect the effects but also helps in understanding the genetic influences on the biological and biochemical pathways that underpin the disease [37]. Two reasons encouraged us to look for interaction between these two genes. First, both the genes are involved in modulating sympathetic and parasympathetic activity [4], [6].Second, these genes are highly associated with phenotypes like adiposity and BMI [14], [15], [24]–[26], [38], which are major risk factors for hypertension [18], [23], [39]–[41]. Our study demonstrated that indeed there was a linear correlation between OR and interacted-genotypes that represented the risk convoking alleles in increasing order. The interaction between the two genes revealed higher OR for risk or lower OR for protection conferring interacted-haplotypes compared to individual respective haplotypes of each gene, thus, supporting the role of epistasis in the regulation of BP [42], [43]. Such interaction studies of *FTO* with other genes are not available. An interaction between *GNB3* and *ACE* however, was documented in EH [44].

With regard to correlation analysis, our findings signified a major contribution of epistasis towards BP phenotypes. The GLM model revealed a significant linear correlation of interacted-genotypes and interacted-haplotypes with clinical parameters e.g., SBP, MAP and BMI. The latter two parameters were increased by >1-fold in the presence of the interacted-haplotypes H_3_+H_c_ and SBP was increased by 2.5-fold in the presence of H_3_+H_d_, suggesting that the interactions of genetic variants played a significant role in determining the observed phenotype [37], [45].

Of consequence, the interactions between genetic variants may modulate the *FTO* expression in metabolically relevant tissues such as hypothalamus, and this may influence subsequent translation of key signaling molecules like *GNB3*; however, this hypothesis needs to be further examined. The other important fact that cannot be ignored is that disease-associated SNPs detected in large-scale association studies are frequently located in noncoding regions, suggesting their involvement in gene regulation [46]; hence, we undertook the TF analysis and observed different sets of transcription factors associating with the risk and protective alleles of both the genes. It is known that the transcriptional regulatory system plays an essential role in controlling numerous biological processes and numerous diseases [46], [47]. Overall, our findings not only supported the available reports but also provided an insight into the interaction of risk variants of *FTO* and *GNB3* in the susceptibility to EH.

Inconsistencies in genetic association studies may be due to, limited statistical power, population stratification and chance of false positive results. To minimize population stratification we recruited the patients and controls from the same region [48]. The likelihood of false positive results was decreased using the Bonferroni’s correction test for multiple testing. As already emphasized, our main aim was to investigate the role of *FTO* and *GNB3* in EH; we adjusted all the results with BMI and other possible confounders to ascertain the direct effect of these genes on hypertension regulation. These adjustments provided evidence of *FTO* and *GNB3* influencing BP. Additional prospective studies on gene-gene interaction are warranted to define the underlying mechanisms in the pathophysiology of EH. The present sample size has been adequate to provide statistically significant associations, but it needs to be tested in larger cohorts with different ethnicities.

In conclusion, the interaction between *FTO* and *GNB3*, through interacted-genotypes and interacted-haplotypes models, markedly has an epistatic effect and associated with altered clinical phenotypes and consequently with EH. The study has also suggested that gene-gene interaction holds robust information about the phenotype beyond analysis of individual SNPs, and thus including interaction between or among genes may improve the predictive accuracy of genetic-clinical correlations.

## Supporting Information

Figure S1 **Linkage disequilibrium (LD) among studied SNPs of** ***FTO*** **and** ***GNB3.*** LD was calculated using Haploview-v4.0 in cases and controls. D’ box shading represents the strength of LD between SNPs. The light shade represents weak LD, whereas dark shade represents strong LD.
(TIF)

Figure S2 **Individual haplotypes of** ***FTO*** **and** ***GNB3*** **in cases and controls.** Total 9 haplotypes of *FTO* were inferred from five SNPs (rs8050136C/A, rs9939609T/A, rs9926289G/A, rs9930506A/G and rs9932754T/C) and 4 haplotypes of *GNB3* from 2 SNPs (rs1129649T/C and rs5443C/T) at overall cutoff frequency of >2%. The symbol *^†^ represents statistically significant risk haplotypes, *FTO* H_3,_ *GNB3* H_c_ and H_d_, (OR = 1.48, 95% CI = 1.13−1.94, *P* = 0.005; OR = 1.74, 95% CI = 1.33−2.26, *P* = 4.36E−05 and OR = 1.79, 95% CI = 1.32−2.43, *P* = 1.79E−04, respectively); whereas, symbol ^#†^ represents, statistically significant protective haplotypes, *FTO* H_4_ and *GNB3* H_a_ (OR = 0.38, 95% CI = 0.23−0.61, *P* = 5.45E−05; OR = 0.49, 95% CI = 0.40−0.61, *P* = 2.12E−11, respectively). *P*-value and odds ratio (OR) were calculated after adjustment for age, gender, BMI, alcohol, smoking, triglyceride and cholesterol using multivariate logistic regression analysis and Bonferroni’s correction for multiple testing.
(TIF)

Figure S3 **Gene-gene interaction between** ***FTO*** **and** ***GNB3*** **.** The gene-gene interaction was looked using Hap Evolution software in case-control haplotypes data. 1, represents major allele and 2, represents minor allele of each SNP. The SNPs *GNB3* rs1129649T/C, rs5443 and *FTO* rs8050136C/T, rs9939609T/A, rs9926289G/A, rs9930506A/G and rs9932754T/C are arranged according to their position on chromosomes. Maximum interaction ratio was observed for minor allele *GNB3* rs5443T and *FTO* rs9932754C. The *P*-value and haplotype risk ratio (HRR) were computed after permutation test.
(TIF)

Figure S4 **Diagrammatic representation of the effects of** ***FTO*** **and** ***GNB3*** **SNPs on transcription factors binding sites.** The upper and lower TF, binding sites with BA represents the protective alleles (marked in black) and risk alleles (marked in red), respectively. The prediction of transcription factor, their binding sites and their binding affinity were performed by online software *TFSEARCH: Searching Transcription Factor Binding Sites,* http://www.rwcp.or.jp/papia/developed by Yutaka Akiyama. TF, transcription factor; BA, binding affinity (%). Flanking sequences in blue represent transcription factor binding sites (TFBS).
(TIF)

Table S1 **Primer sequences, normal and SNapShot PCR cycling conditions used for genotyping of** ***FTO*** **studied polymorphisms.**
(DOC)

Table S2 **Primers, RFLP and SNapShot PCR cycling conditions for genotyping** ***GNB3*** **polymorphisms.**
(DOC)

Table S3 **Goodness-of-fit test for observed and expected genotypes distribution of** ***FTO*** **and** ***GNB3*** **polymorphisms in patients and controls.** Comparison between observed and expected frequencies was performed by an epidemiologic data management and analysis package (EPIINFO) ver.6
(DOC)

Table S4 **Comparison of the** ***FTO*** **and** ***GNB3*** **studied alleles frequencies with Hapmap population.** *P*-values were calculated using EPIINFO ver.6 (Center for Disease Control, Atlanta, Georgia, USA) software. Indian (IND) population was used as reference.
(DOC)

Table S5 **Genotypes and allele distribution of the** ***FTO*** **and** ***GNB3*** **polymorphisms in controls and patients.** *P* value, χ^2^ and odds ratio (OR) were calculated using multivariate logistic regression analysis after adjustment for age, gender, BMI, smoking, alcohol, triglycerides and cholesterol.
(DOC)

## References

[1] Kearney PM, Whelton M, Reynolds K, Muntner P, Whelton PK, et al (2005) Global burden of hypertension: analysis of worldwide data. Lancet 365: 217–223. 15652604 10.1016/S0140-6736(05)17741-1

[2] Joyner MJ, Charkoudian N, Wallin BG (2008) A sympathetic view of the sympathetic nervous system and human blood pressure regulation. Exp Physiol 93: 715–724. 18326553 10.1113/expphysiol.2007.039545 PMC3433836

[3] Xia H, Suda S, Bindom S, Feng Y, Gurley SB, et al (2011) ACE2-Mediated reduction of oxidative stress in the central nervous system is associated with improvement of autonomic function. PLoS One 6: e22682. 21818366 10.1371/journal.pone.0022682 PMC3144922

[4] Guyenet PG (2006) The sympathetic control of blood pressure. Nat Rev Neurosci 7: 335–346. 16760914 10.1038/nrn1902

[5] Bunnett NW, Cottrell GS (2010) Trafficking and signaling of G protein-coupled receptors in the nervous system: implications for disease and therapy. CNS Neurol Disord Drug Targets 9: 539–556. 20632972 10.2174/187152710793361621

[6] Zolk O, Kouchi I, Schnabel P, Bohm M (2000) Heterotrimeric G proteins in heart disease. Can J Physiol Pharmacol 78: 187–198. 10721810

[7] Mitchell BD, Kammerer CM, Blangero J, Mahaney MC, Rainwater DL, et al (1996) Genetic and environmental contributions to cardiovascular risk factors in Mexican Americans. The San Antonio Family Heart Study. Circulation 94: 2159–2170. 8901667 10.1161/01.cir.94.9.2159

[8] Hong Y, de FU, Heller DA, McClearn GE, Pedersen N (1994) Genetic and environmental influences on blood pressure in elderly twins. Hypertension 24: 663–670. 7995622 10.1161/01.hyp.24.6.663

[9] Harrap SB, Stebbing M, Hopper JL, Hoang HN, Giles GG (2000) Familial patterns of covariation for cardiovascular risk factors in adults: The victorian family heart study. Am J Epidemiol 152: 704–715. 11052548 10.1093/aje/152.8.704

[10] Yang J, Loos RJ, Powell JE, Medland SE, Speliotes EK, et al (2012) FTO genotype is associated with phenotypic variability of body mass index. Nature 490: 267–272. 22982992 10.1038/nature11401 PMC3564953

[11] Fall T, Ingelsson E (2012) Genome-wide association studies of obesity and metabolic syndrome. Mol Cell Endocrinol. S0303-7207(12)00413-3. 10.1016/j.mce.2012.08.018 22963884

[12] Peng S, Zhu Y, Xu F, Ren X, Li X, et al (2011) FTO gene polymorphisms and obesity risk: a meta-analysis. BMC Med 9: 71. 21651756 10.1186/1741-7015-9-71 PMC3118373

[13] Niu W, Qi Y (2011) Association of alpha-adducin and G-protein beta3 genetic polymorphisms with hypertension: a meta-analysis of Chinese populations. PLoS One 6: e17052. 21364877 10.1371/journal.pone.0017052 PMC3045422

[14] Wen W, Cho YS, Zheng W, Dorajoo R, Kato N, et al (2012) Meta-analysis identifies common variants associated with body mass index in east Asians. Nat Genet 44: 307–311. 22344219 10.1038/ng.1087 PMC3288728

[15] Pemberton TJ, Mehta NU, Witonsky D, Di RA, Allayee H, et al (2008) Prevalence of common disease-associated variants in Asian Indians. BMC Genet 9: 13. 18248681 10.1186/1471-2156-9-13 PMC2267478

[16] Gerken T, Girard CA, Tung YC, Webby CJ, Saudek V, et al (2007) The obesity-associated FTO gene encodes a 2-oxoglutarate-dependent nucleic acid demethylase. Science 318: 1469–1472. 17991826 10.1126/science.1151710 PMC2668859

[17] Hamet P, Merlo E, Seda O, Broeckel U, Tremblay J, et al (2005) Quantitative founder-effect analysis of French Canadian families identifies specific loci contributing to metabolic phenotypes of hypertension. Am J Hum Genet 76: 815–832. 15800845 10.1086/430133 PMC1199371

[18] Pausova Z, Syme C, Abrahamowicz M, Xiao Y, Leonard GT, et al (2009) A common variant of the FTO gene is associated with not only increased adiposity but also elevated blood pressure in French Canadians. Circ Cardiovasc Genet 2: 260–269. 20031594 10.1161/CIRCGENETICS.109.857359

[19] Neves SR, Ram PT, Iyengar R (2002) G protein pathways. Science 296: 1636–1639. 12040175 10.1126/science.1071550

[20] Siffert W, Rosskopf D, Siffert G, Busch S, Moritz A, et al (1998) Association of a human G-protein beta3 subunit variant with hypertension. Nat Genet 18: 45–48. 9425898 10.1038/ng0198-45

[21] Siffert W (2003) G-protein beta3 subunit 825T allele and hypertension. Curr Hypertens Rep 5: 47–53. 12530935 10.1007/s11906-003-0010-4

[22] Bagos PG, Elefsinioti AL, Nikolopoulos GK, Hamodrakas SJ (2007) The GNB3 C825T polymorphism and essential hypertension: a meta-analysis of 34 studies including 14,094 cases and 17,760 controls. J Hypertens 25: 487–500. 17278960 10.1097/HJH.0b013e328011db24

[23] Timpson NJ, Harbord R, Davey SG, Zacho J, Tybjaerg-Hansen A, et al (2009) Does greater adiposity increase blood pressure and hypertension risk?: Mendelian randomization using the FTO/MC4R genotype. Hypertension 54: 84–90. 19470880 10.1161/HYPERTENSIONAHA.109.130005

[24] Chang YC, Liu PH, Lee WJ, Chang TJ, Jiang YD, et al (2008) Common variation in the fat mass and obesity-associated (FTO) gene confers risk of obesity and modulates BMI in the Chinese population. Diabetes 57: 2245–2252. 18487448 10.2337/db08-0377 PMC2494679

[25] Souza RP, De LV, Muscettola G, Rosa DV, de BA, et al (2008) Association of antipsychotic induced weight gain and body mass index with GNB3 gene: a meta-analysis. Prog Neuropsychopharmacol Biol Psychiatry 32: 1848–1853. 18793692 10.1016/j.pnpbp.2008.08.014

[26] Klenke S, Kussmann M, Siffert W (2011) The GNB3 C825T polymorphism as a pharmacogenetic marker in the treatment of hypertension, obesity, and depression. Pharmacogenet Genomics 21: 594–606. 21709600 10.1097/FPC.0b013e3283491153

[27] Stephens M, Smith NJ, Donnelly P (2001) A new statistical method for haplotype reconstruction from population data. Am J Hum Genet 68: 978–989. 11254454 10.1086/319501 PMC1275651

[28] Barrett JC, Fry B, Maller J, Daly MJ (2005) Haploview: analysis and visualization of LD and haplotype maps. Bioinformatics 21: 263–265. 15297300 10.1093/bioinformatics/bth457

[29] Hahn LW, Ritchie MD, Moore JH (2003) Multifactor dimensionality reduction software for detecting gene-gene and gene-environment interactions. Bioinformatics 19: 376–382. 12584123 10.1093/bioinformatics/btf869

[30] Potter MA, De Jong KA (2000) Cooperative coevolution: an architecture for evolving coadapted subcomponents. Evol Comput 8: 1–29. 10753229 10.1162/106365600568086

[31] Ahmad T, Chasman DI, Mora S, Pare G, Cook NR, et al (2010) The fat-mass and obesity-associated (FTO) gene, physical activity, and risk of incident cardiovascular events in white women. Am Heart J 160: 1163–1169. 21146673 10.1016/j.ahj.2010.08.002 PMC3058560

[32] Sentinelli F, Incani M, Coccia F, Capoccia D, Cambuli VM, et al (2012) Association of FTO polymorphisms with early age of obesity in obese Italian subjects. Exp Diabetes Res. 2012: 872176. 10.1155/2012/872176 PMC3290805 22454631

[33] Nejatizadeh A, Kumar R, Stobdan T, Qadar Pasha MA (2011) Association of GNB3 C825T polymorphism with plasma electrolyte balance and susceptibility to hypertension. Genet Mol Biol 34: 553–556. 22215956 10.1590/S1415-47572011005000052 PMC3229107

[34] Wenzel RR, Siffert W, Bruck H, Philipp T, Schafers RF (2002) Enhanced vasoconstriction to endothelin-1, angiotensin II and noradrenaline in carriers of the GNB3 825T allele in the skin microcirculation. Pharmacogenetics 12: 489–495. 12172218 10.1097/00008571-200208000-00010

[35] Kelly TN, Rice TK, Gu D, Hixson JE, Chen J, et al (2009) Novel genetic variants in the alpha-adducin and guanine nucleotide binding protein beta-polypeptide 3 genes and salt sensitivity of blood pressure. Am J Hypertens 22: 985–992. 19574959 10.1038/ajh.2009.118 PMC2882159

[36] Cordell HJ (2009) Detecting gene-gene interactions that underlie human diseases. Nat Rev Genet 10: 392–404. 19434077 10.1038/nrg2579 PMC2872761

[37] Moore JH (2003) The ubiquitous nature of epistasis in determining susceptibility to common human diseases. Hum Hered 56: 73–82. 14614241 10.1159/000073735

[38] Dina C, Meyre D, Gallina S, Durand E, Korner A, et al (2007) Variation in FTO contributes to childhood obesity and severe adult obesity. Nat Genet 39: 724–726. 17496892 10.1038/ng2048

[39] Mamun AA, Lawlor DA, O’Callaghan MJ, Williams GM, Najman JM (2005) Effect of body mass index changes between ages 5 and 14 on blood pressure at age 14: findings from a birth cohort study. Hypertension 45: 1083–1087. 15897371 10.1161/01.HYP.0000166720.18319.51

[40] Neter JE, Stam BE, Kok FJ, Grobbee DE, Geleijnse JM (2003) Influence of weight reduction on blood pressure: a meta-analysis of randomized controlled trials. Hypertension 42: 878–884. 12975389 10.1161/01.HYP.0000094221.86888.AE

[41] Jones DW (1996) Body weight and blood pressure. Effects of weight reduction on hypertension. Am J Hypertens 9: 50s–54s. 8862237 10.1016/0895-7061(96)00183-5

[42] Rana BK, Insel PA, Payne SH, Abel K, Beutler E, et al (2007) Population-based sample reveals gene-gender interactions in blood pressure in White Americans. Hypertension 49: 96–106. 17159089 10.1161/01.HYP.0000252029.35106.67

[43] Zhao Q, Wang L, Yang W, Chen S, Huang J, et al (2008) Interactions among genetic variants from contractile pathway of vascular smooth muscle cell in essential hypertension susceptibility of Chinese Han population. Pharmacogenet Genomics 18: 459–466. 18496125 10.1097/FPC.0b013e3282f97fb2

[44] Bae Y, Park C, Han J, Hong YJ, Song HH, et al (2007) Interaction between GNB3 C825T and ACE I/D polymorphisms in essential hypertension in Koreans. J Hum Hypertens 21: 159–166. 17066084 10.1038/sj.jhh.1002110

[45] Newton-Cheh C, Hirschhorn JN (2005) Genetic association studies of complex traits: design and analysis issues. Mutat Res 573: 54–69. 15829237 10.1016/j.mrfmmm.2005.01.006

[46] Ameur A, Rada-Iglesias A, Komorowski J, Wadelius C (2009) Identification of candidate regulatory SNPs by combination of transcription-factor-binding site prediction, SNP genotyping and haploChIP. Nucleic Acids Res 37: e85. 19451166 10.1093/nar/gkp381 PMC2709586

[47] Vaquerizas JM, Kummerfeld SK, Teichmann SA, Luscombe NM (2009) A census of human transcription factors: function, expression and evolution. Nat Rev Genet 10: 252–263. 19274049 10.1038/nrg2538

[48] Indian Genome Variation Consortium (2008) Genetic landscape of the people of India: a canvas for disease gene exploration. J Genet 87: 3–20. 18560169 10.1007/s12041-008-0002-x

