# Supplementary figures and images for: Interactions between the FTO and GNB3 Genes Contribute to Varied Clinical Phenotypes in Hypertension
Source: PLoS One. 2013 May 14;8(5):e63934. doi: 10.1371/journal.pone.0063934 (PMC3653800; doi:10.1371/journal.pone.0063934)

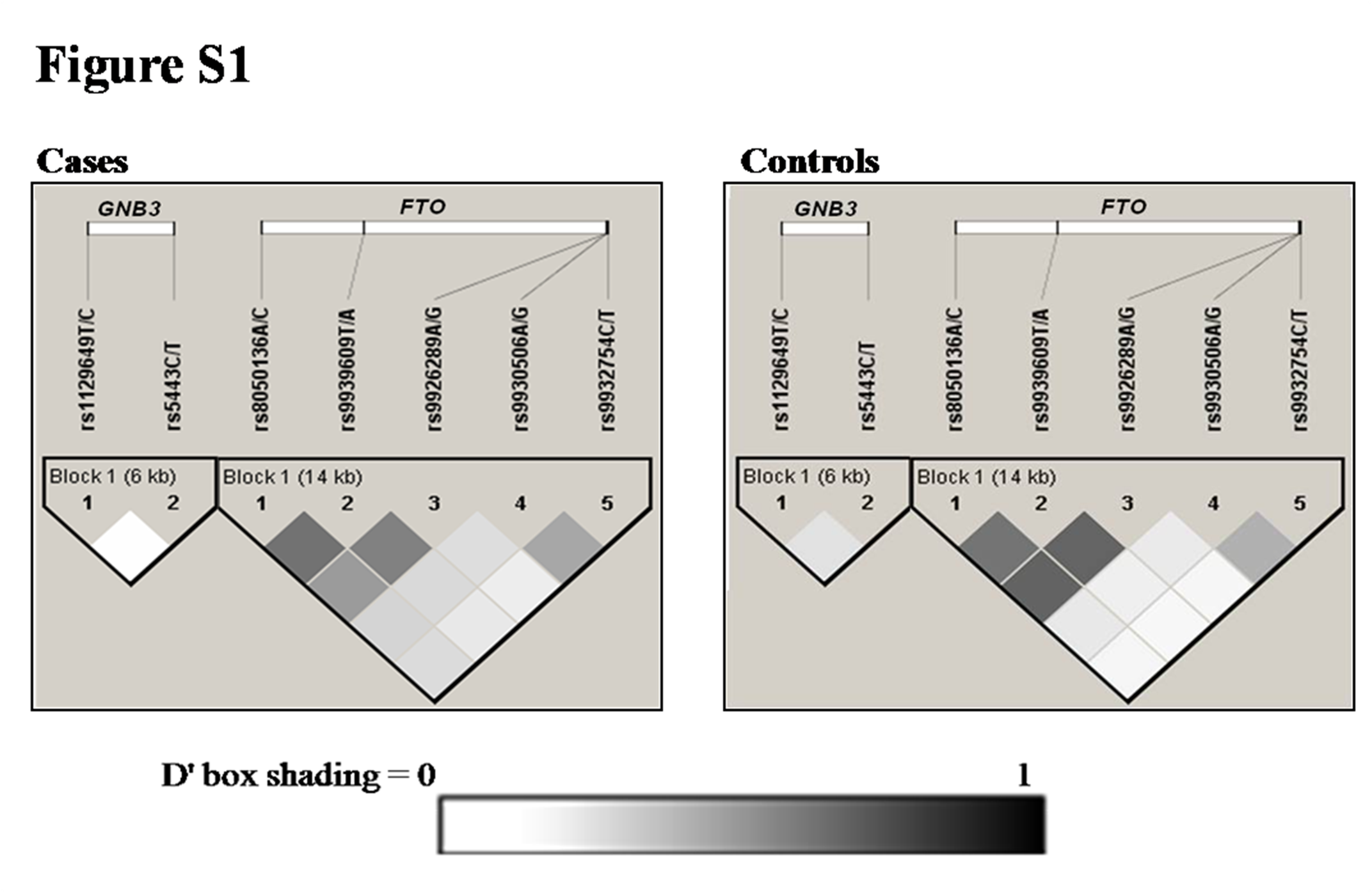

Supplement: Figure S1 — Linkage disequilibrium (LD) among studied SNPs of FTO and GNB3. LD was calculated using Haploview-v4.0 in cases and controls. D’ box shading represents the strength of LD between SNPs. The light shade represents weak LD, whereas dark shade represents strong LD. (TIF) [file pone.0063934.s001.tif]

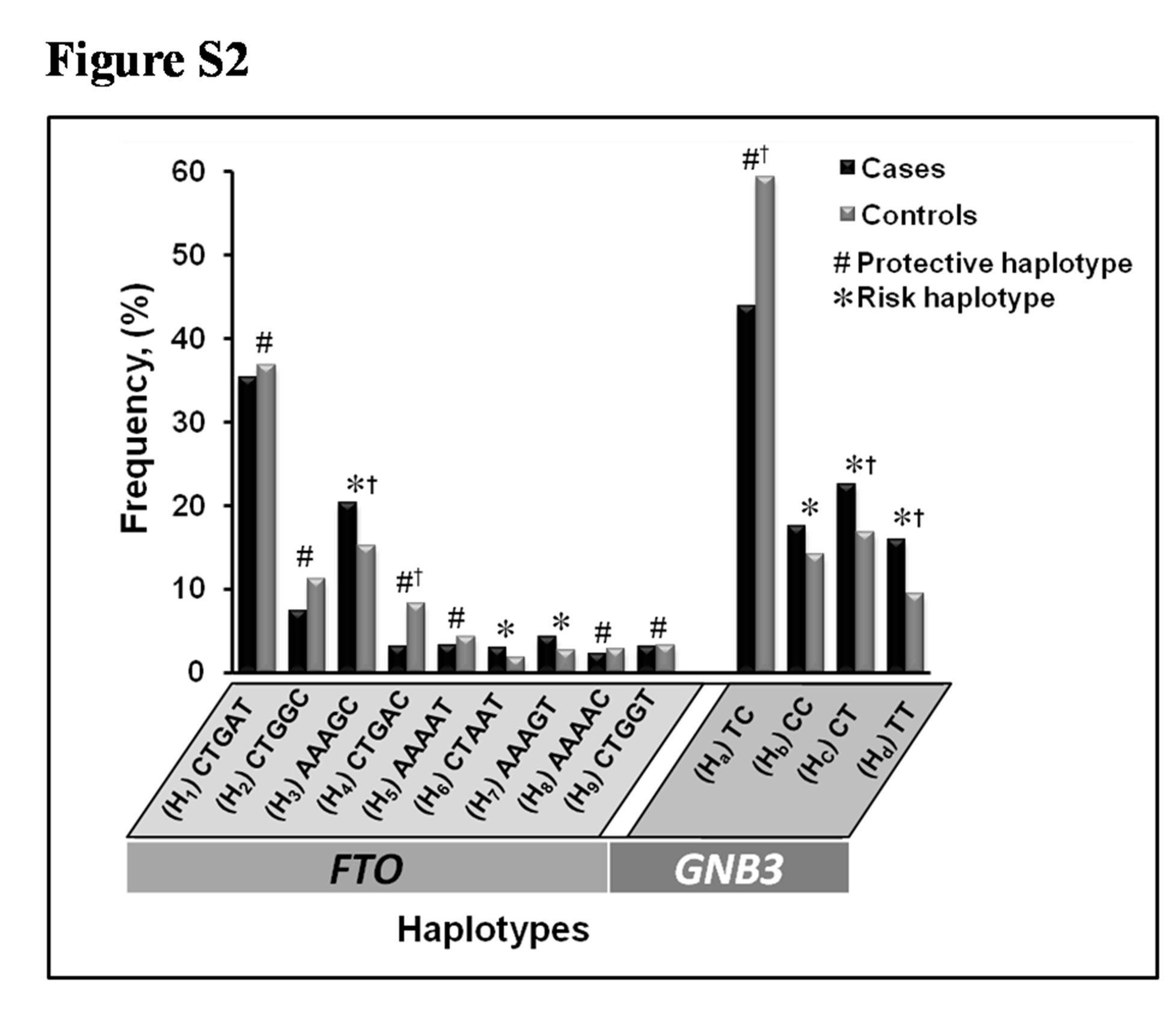

Supplement: Figure S2 — Individual haplotypes of FTO and GNB3 in cases and controls. Total 9 haplotypes of FTO were inferred from five SNPs (rs8050136C/A, rs9939609T/A, rs9926289G/A, rs9930506A/G and rs9932754T/C) and 4 haplotypes of GNB3 from 2 SNPs (rs1129649T/C and rs5443C/T) at overall cutoff frequency of >2%. The symbol *† represents statistically significant risk haplotypes, FTO H3, GNB3 Hc and Hd, (OR = 1.48, 95% CI = 1.13−1.94, P = 0.005; OR = 1.74, 95% CI = 1.33−2.26, P = 4.36E−05 and OR = 1.79, 95% CI = 1.32−2.43, P = 1.79E−04, respectively); whereas, symbol #† represents, statistically significant protective haplotypes, FTO H4 and GNB3 Ha (OR = 0.38, 95% CI = 0.23−0.61, P = 5.45E−05; OR = 0.49, 95% CI = 0.40−0.61, P = 2.12E−11, respectively). P-value and odds ratio (OR) were calculated after adjustment for age, gender, BMI, alcohol, smoking, triglyceride and cholesterol using multivariate logistic regression analysis and Bonferroni’s correction for multiple testing. (TIF) [file pone.0063934.s002.tif]

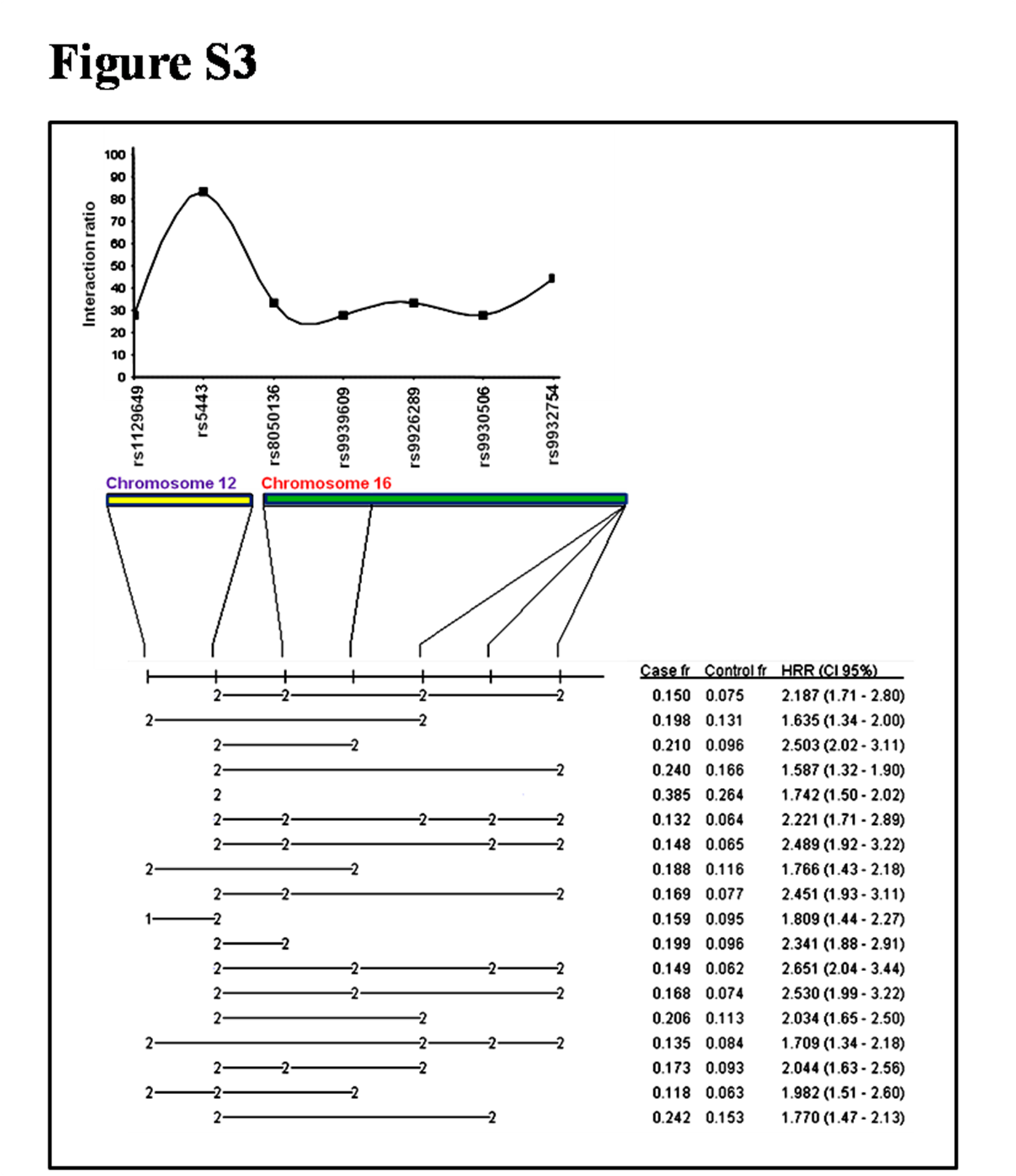

Supplement: Figure S3 — Gene-gene interaction between FTO and GNB3 . The gene-gene interaction was looked using Hap Evolution software in case-control haplotypes data. 1, represents major allele and 2, represents minor allele of each SNP. The SNPs GNB3 rs1129649T/C, rs5443 and FTO rs8050136C/T, rs9939609T/A, rs9926289G/A, rs9930506A/G and rs9932754T/C are arranged according to their position on chromosomes. Maximum interaction ratio was observed for minor allele GNB3 rs5443T and FTO rs9932754C. The P-value and haplotype risk ratio (HRR) were computed after permutation test. (TIF) [file pone.0063934.s003.tif]

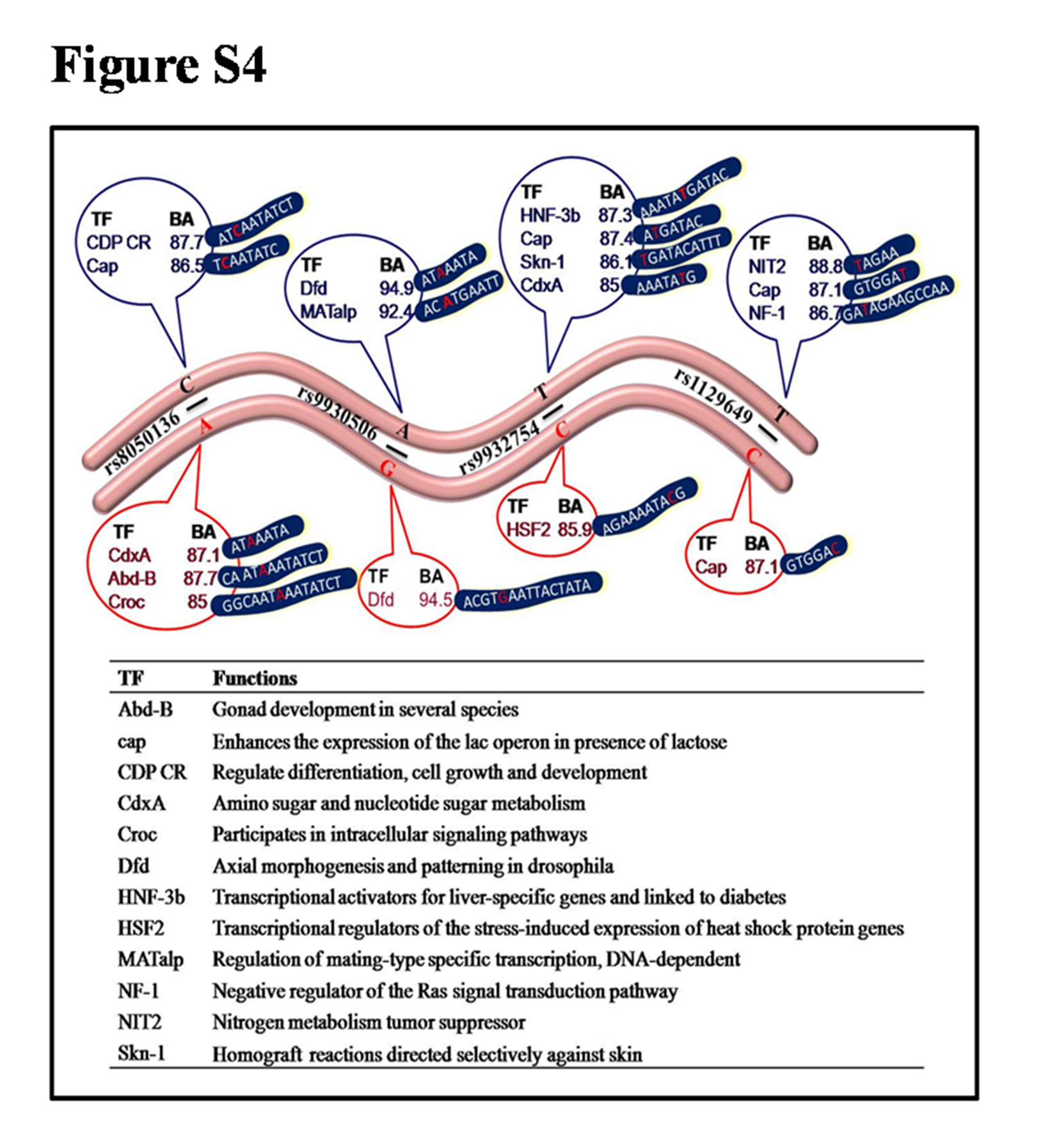

Supplement: Figure S4 — Diagrammatic representation of the effects of FTO and GNB3 SNPs on transcription factors binding sites. The upper and lower TF, binding sites with BA represents the protective alleles (marked in black) and risk alleles (marked in red), respectively. The prediction of transcription factor, their binding sites and their binding affinity were performed by online software TFSEARCH: Searching Transcription Factor Binding Sites, http://www.rwcp.or.jp/papia/developed by Yutaka Akiyama. TF, transcription factor; BA, binding affinity (%). Flanking sequences in blue represent transcription factor binding sites (TFBS). (TIF) [file pone.0063934.s004.tif]
