# Supplementary material for: Interactions between the FTO and GNB3 Genes Contribute to Varied Clinical Phenotypes in Hypertension
Source: PLoS One. 2013 May 14;8(5):e63934. doi: 10.1371/journal.pone.0063934 (PMC3653800; doi:10.1371/journal.pone.0063934)
Supplement: Table S1 — Primer sequences, normal and SNapShot PCR cycling conditions used for genotyping of FTO studied polymorphisms. (DOC) [file pone.0063934.s005.doc]

**Table S1: Primer sequences, normal and SNapShot PCR cycling conditions used for genotyping of studied *FTO* SNPs**

| **rs ID** | **SNP** | **Primer sequences** | **Normal PCR condition** | **SNapShot PCR condition** | **Visualized peaks** |
| --- | --- | --- | --- | --- | --- |
| s8050136 | C/A | F 5' GAG TCC ATA CCA ACC AAG GTC C 3' | ID 94°C 4', D 94°C 45'', | D 96°C 10'', A 54°C 5'', | CC=Black |
|  |  | R 5' CCA TGA GTC CAT CTC TAC AG 3' | A 61°C 30'', E 72°C 45'', | E 60°C 10''; 35 Cy | AA=Green |
|  |  | S 5' TTG CCC ACT GTG GCA AT 3' | 32cy, FE 72°C 10' |  | CA=Black & Green |
| rs9939609 | T /A | F 5' GCA TGG ATT CAA TGC AAA ATG GC 3' | ID 94°C 4', D 94°C 30'', | D 96°C 10'', A 55°C 5'', | TT=Red |
|  |  | R 5' AGC CCA AGG ATG GTG TTT CTA AGG A | A 60.2°C 30'', E 72°C 45'', | E 60°C 10''; 35 Cy | AA=Green |
|  |  | S 5' GTG ATG CAC TTG GAT AGT C 3' | 35cy, FE 72°C 10' |  | TA=Red & Green |
| rs9926289 | G/A | F 5' GCA TGG ATT CAA TGC AAA ATG GC 3' | ID 94°C 4', D 94°C 30'', | D 96°C 10'', A 54°C 5'', | GG=Blue |
|  |  | R 5' AGC CCA AGG ATG GTG TTT CTA AGG A 3' | A 60.2°C 30'', E 72°C 45'', |  | AA=Green |
|  |  | S 5' GTC TGA ATT ATT ATT CTA G 3' | 35cy, FE 72°C 10' | E 60°C 10''; 35 Cy | GA=Blue & Green |
| rs9930506 | A/G | F 5' TTA GGG ACA CAA AAA GGG AC 3' | ID 94°C 4', D 94°C 45'', | D 96°C 10'', A 54°C 5'', | AA=Green |
|  |  | R 5' GTA GCT GCC CAG AAC CAC TAG 3' | A 58.6°C 30'', E 72°C 45'', | E 60°C 10''; 35 Cy | GG=Blue |
|  |  | S 5' CTT AGA TAT TAG TAA TTC A 3' | 32cy, FE 72°C 10' |  | AG=Green & Blue |
| rs9932754 | T/C | F 5' TTA GGG ACA CAA AAA GGG AC 3' | ID 94°C 4', D 94°C 45'', | D 96°C 10'', A 54°C 5'', | TT=Red |
|  |  | R 5' GTA GCT GCC CAG AAC CAC TAG 3' | A 58.6°C 30'', E 72°C 45'', | E 60°C 10''; 35 Cy | CC=Black |
|  |  | S 5' ACT AAT ATC TAA GAA AAT A 3' | 32cy, FE 72°C 10' |  | TC=Red & Black |
| rs9933040 | A/T | F 5' TTA GGG ACA CAA AAA GGG AC 3' | ID 94°C 4', D 94°C 45'', | D 96°C 10'', A 59°C 5'', | AA=Green |
|  |  | R 5' GTA GCT GCC CAG AAC CAC TAG 3' | A 58.6°C 30'', E 72°C 45'', | E 60°C 10''; 35 Cy | TT=Red |
|  |  | S 5' TCC TTC TCT CCC CTA GTG G 3' | 32cy, FE 72°C 10' |  | AT=Green & Red |
| rs62033414 | C/G | F 5' TTA GGG ACA CAA AAA GGG AC 3' | ID 94°C 4', D 94°C 45'', | D 96°C 10'', A 61°C 5'', | CC=Black |
|  |  | R 5' GTA GCT GCC CAG AAC CAC TAG 3' | A 58.6°C 30'', E 72°C 45'', | E 60°C 10''; 35 Cy | GG=Blue |
|  |  | S 5' CCC TAG TGG ATC TGG GCA G 3' | 32cy, FE 72°C 10' |  | CG=Black & Blue |
| rs16952624 | C/T | F 5' GTG CAC TGA CTG GTG GTG TC 3’ | ID 94°C 4', D 94°C 45'', | D 96°C 10'', A 61°C 5'', | CC=Black |
|  |  | R 5' TCC TGG CTA TAC CCA TCA CC 3' | A 67°C 30'', E 72°C 45'', | E 60°C 10''; 35 Cy | TT=Red |
|  |  | S 5' CAT GGC TCA ACT GGA AG 3' | 30cy, FE 72°C 10' |  | CT=Black & Red |
| rs16953075 | C/T | F 5' GAC CTG CAT CAC CCT TTC AT 3' | ID 94°C 4', D 94°C 45'', | D 96°C 10'', A 55°C 5'', | CC=Black |
|  |  | R 5' CTC CAA CCC TGT TGC ACA TTC C 3' | A 64°C 45'', E 72°C 45'', | E 60°C 10''; 35 Cy | TT=Red |
|  |  | S 5' ATT AGT TCT CAA AGT CCT 3' | 30cy, FE 72°C 10' |  | CT=Black & Red |

F= forward; R= reverse; S= SNapShot primer; ID= initial denaturation; D= denaturation; A= annealing; E= extension; cy= cycles; FE= final extension
