# Supplementary material for: Interactions between the FTO and GNB3 Genes Contribute to Varied Clinical Phenotypes in Hypertension
Source: PLoS One. 2013 May 14;8(5):e63934. doi: 10.1371/journal.pone.0063934 (PMC3653800; doi:10.1371/journal.pone.0063934)
Supplement: Table S2 — Primers, RFLP and SNapShot PCR cycling conditions for genotyping GNB3 polymorphisms. (DOC) [file pone.0063934.s006.doc]

**Table S2:** **Primers, RFLP and SNapShot PCR cycling conditions for genotyping *GNB3* SNPs**

| **rs ID** | **SNP** | **Primer sequences** | **Cycling condition** | **SNapShot condition** | | **Visualized peaks** |
| --- | --- | --- | --- | --- | --- | --- |
| rs5443 | C/T | F 5' TGA CCC ACT TGC CAC CCG TGC 3' | ID 95°C 4', D 95°C 30'', | D 96°C 10'', A 58°C 5'', | | CC=Black |
|  |  | R 5' GCA GCA GCC AGG GCT GGC 3' | A 63°C 45'', E 72°C 45'', | E 60°C 10''; 35 Cy | | TT=Red |
|  |  | S 5' CTG AGG GAG AAG GCC AC 3' | 35cy, FE 72°C 7' |  | | CT=Black & Red |
|  |  |  |  | RestrictionEnzymes | Digestion Condition | Visualized bands (bp) |
| rs1129649 | T/C | F 5' GGG TAG GAA GGG ATG TGG TT 3' | ID 94°C 4', D 94°C 30'', | *HYP81* | 37°C | TT =446 |
|  |  | R 5' AGA TGG TGG GGG TGC AGG GA 3' | A 68°C 45'', E 72°C 45'', | Tango Buffer | overnight | TC = 446, 295, 151 |
|  |  |  | 35cy, FE 72°C 10' |  |  | CC= 295, 151 |

F= forward; R= reverse; S= SNapShot primer; ID= initial denaturation; D= denaturation; A= annealing; E= extension; cy= cycles; FE= final extension.
