# Supplementary material for: Interactions between the FTO and GNB3 Genes Contribute to Varied Clinical Phenotypes in Hypertension
Source: PLoS One. 2013 May 14;8(5):e63934. doi: 10.1371/journal.pone.0063934 (PMC3653800; doi:10.1371/journal.pone.0063934)
Supplement: Table S3 — Goodness-of-fit test for observed and expected genotypes distribution of FTO and GNB3 polymorphisms in patients and controls. Comparison between observed and expected frequencies was performed by an epidemiologic data management and analysis package (EPIINFO) ver.6 (DOC) [file pone.0063934.s007.doc]

| **Table S3:** **Goodness-of-fit test for observed and expected genotypes distribution of *FTO* and *GNB3* SNPs in patients and controls** | | | | | | | | |
| --- | --- | --- | --- | --- | --- | --- | --- | --- |
| **Gene** | **SNP** | | **Genotype** | **Controls** | |  | **Patients** | |
|  |  | |  | **Observed** | **Expected** |  | **Observed** | **Expected** |
| *FTO* | rs8050136C/A | | rs8050136CC | 47% | 46% |  | 37% | 37% |
|  |  | | rs8050136CA | 42% | 44% |  | 47% | 48% |
|  |  | | rs8050136AA | 11% | 10% |  | 16% | 15% |
|  |  | | χ^2^ | 0.21 |  |  | 0.09 |  |
|  |  | | *P* value | 0.899 |  |  | 0.957 |  |
|  |  | |  |  |  |  |  |  |
|  | rs9939609T/A | | rs9939609TT | 47% | 47% |  | 37% | 36% |
|  |  | | rs9939609TA | 43% | 43% |  | 47% | 48% |
|  |  | | rs9939609AA | 10% | 10% |  | 16% | 16% |
|  |  | | χ^2^ | 0.00 |  |  | 0.05 |  |
|  |  | | *P* value | 1.000 |  |  | 0.976 |  |
|  |  | |  |  |  |  |  |  |
|  | rs9926289G/A | | rs9926289GG | 41% | 41% |  | 32% | 33% |
|  |  | | rs9926289GA | 46% | 46% |  | 51% | 49% |
|  |  | | rs9926289AA | 13% | 13% |  | 17% | 18% |
|  |  | | χ^2^ | 0.00 |  |  | 0.17 |  |
|  |  | | *P* value | 1.000 |  |  | 0.920 |  |
|  |  | |  |  |  |  |  |  |
|  | rs9930506A/G | | rs9930506AA | 38% | 35% |  | 30% | 29% |
|  |  | | rs9930506AG | 43% | 48% |  | 49% | 50% |
|  |  | | rs9930506GG | 19% | 17% |  | 21% | 21% |
|  |  | | χ^2^ | 1.01 |  |  | 0.05 |  |
|  |  | | *P* value | 0.602 |  |  | 0.973 |  |
|  |  | |  |  |  |  |  |  |
|  | rs9932754T/C | | rs9932754TT | 31% | 28% |  | 34% | 33% |
|  |  | | rs9932754TC | 44% | 50% |  | 47% | 49% |
|  |  | | rs9932754CC | 25% | 22% |  | 19% | 18% |
|  |  | | χ^2^ | 1.45 |  |  | 0.17 |  |
|  |  | | *P* value | 0.484 |  |  | 0.920 |  |
|  | |  | | | | | | |
| *GNB3* | rs1129649T/C | | rs1129649TT | 48% | 47% |  | 31% | 36% |
|  |  | | rs1129649TC | 42% | 43% |  | 57% | 48% |
|  |  | | rs1129649CC | 10% | 10% |  | 12% | 16% |
|  |  | | χ2 | 0.04 |  |  | 3.38 |  |
|  |  | | *P* value | 0.978 |  |  | 0.184 |  |
|  |  | |  |  |  |  |  |  |
|  | rs5443C/T | | rs5443CC | 57% | 54% |  | 38% | 38% |
|  |  | | rs5443CT | 36% | 39% |  | 47% | 47% |
|  |  | | rs5443TT | 07% | 07% |  | 15% | 15% |
|  |  | | χ2 | 0.40 |  |  | 0.00 |  |
|  |  | | *P* value | 0.820 |  |  | 1.000 |  |
