# Supplementary material for: Interactions between the FTO and GNB3 Genes Contribute to Varied Clinical Phenotypes in Hypertension
Source: PLoS One. 2013 May 14;8(5):e63934. doi: 10.1371/journal.pone.0063934 (PMC3653800; doi:10.1371/journal.pone.0063934)
Supplement: Table S4 — Comparison of the FTO and GNB3 studied alleles frequencies with Hapmap population. P-values were calculated using EPIINFO ver.6 (Center for Disease Control, Atlanta, Georgia, USA) software. Indian (IND) population was used as reference. (DOC) [file pone.0063934.s008.doc]

**Table S4: Comparison of the *FTO* and *GNB3* studied SNPs allele frequencies with HapMap population**

| **Gene** | **SNP** | **Alleles** | **Distribution (%)** | | | | | |
| --- | --- | --- | --- | --- | --- | --- | --- | --- |
|  |  |  | **IND** | **CEU** | **YRI** | **CHB** | **JPT** | **ASW** |
| ***FTO*** | rs8050136 | C | 65 | 54 | 52 | 86 | 82 | 55 |
|  |  | A | 35 | 46 | 48 | 14 | 18 | 45 |
|  |  |  | *P*-value | 0.113 | 0.06 | 0.0006 | 0.006 | 0.149 |
|  | rs9939609 | T | 64 | 54 | 47 | 85 | 81 | 51 |
|  |  | A | 36 | 46 | 53 | 15 | 19 | 49 |
|  |  |  | *P* -value | 0.151 | 0.016 | 0.0006 | 0.007 | 0.063 |
|  | rs9926289 | G | 61 | NA | 46 | 88 | 83 | NA |
|  |  | A | 39 |  | 54 | 12 | 17 |  |
|  |  |  | *P* -value | - | 0.033 | 0.00001 | 0.0005 | - |
|  | rs9930506 | A | 57 | 52 | 80 | 79 | 77 | 79 |
|  |  | G | 43 | 48 | 20 | 21 | 23 | 21 |
|  |  |  | *P* -value | 0.478 | 0.0005 | 0.0008 | 0.003 | 0.0008 |
|  | rs9932754 | T | 55 | 52 | NA | NA | NA | NA |
|  |  | C | 45 | 48 |  |  |  |  |
|  |  |  | *P* -value | 0.671 | - | - | - | - |
| ***GNB3*** | rs1129649 | T | 65 | 64 | 57 | 67 | 64 | 55 |
|  |  | C | 35 | 36 | 43 | 33 | 36 | 45 |
|  |  |  | *P* -value | 0.882 | 0.246 | 0.765 | 0.882 | 0.149 |
|  | rs5443 | C | 68 | 62 | 9 | 66 | 60 | NA |
|  |  | T | 32 | 38 | 91 | 34 | 40 |  |
|  |  |  | *P* -value | 0.373 | <0.0001 | 0.763 | 0.238 | - |
| IND, CEU, YRI, CHB, JPT and ASW represent Indian (this study), Caucasian, African, Chinese, Japanese and African-American population, respectively. | | | | | | | | |
