# Supplementary material for: Interactions between the FTO and GNB3 Genes Contribute to Varied Clinical Phenotypes in Hypertension
Source: PLoS One. 2013 May 14;8(5):e63934. doi: 10.1371/journal.pone.0063934 (PMC3653800; doi:10.1371/journal.pone.0063934)
Supplement: Table S5 — Genotypes and allele distribution of the FTO and GNB3 polymorphisms in controls and patients. P value, χ2 and odds ratio (OR) were calculated using multivariate logistic regression analysis after adjustment for age, gender, BMI, smoking, alcohol, triglycerides and cholesterol. (DOC) [file pone.0063934.s009.doc]

**Table S5: Genotypes and allele distribution of the *FTO* and *GNB3* SNPs in controls and patients**

| **Gene/ SNP** | **Genotype/allele** | **Controls Patients** | | **Biostatistical analysis** | | |
| --- | --- | --- | --- | --- | --- | --- |
| ***FTO*** |  | **n, (distribution, %)** | | **χ^2^** | ***P* value** | **OR (95%CI)** |
| rs8050136C/A | rs8050136CC | 350 (47%) | 205 (37%) | – | – | Reference |
|  | rs8050136CA | 320 (42%) | 259 (47%) | 4.90 | 0.027 | 1.42(1.04–1.93) |
|  | rs8050136AA | 80 (11%) | 86 (16%) | 3.78 | 0.052 | 1.59(1.00–2.52) |
|  | rs8050136CA+AA | 400 (53%) | 345(63%) | 6.24 | 0.012 | 1.45(1.08–1.95) |
|  | rs8050136C | 1020 (68%) | 669 (61%) | – | – | Reference |
|  | rs8050136A | 480 (32%) | 431 (39%) | 6.04 | 0.014 | 1.31(1.06–1.62) |
|  |  |  |  |  |  |  |
| rs9939609T/A | rs9939609TT | 355 (47%) | 200 (37%) | – | – | Reference |
|  | rs9939609TA | 320 (43%) | 260 (47%) | 4.33 | 0.037 | 1.39(1.02–1.89) |
|  | rs9939609AA | 75 (10%) | 90 (16%) | 8.43 | 0.005 | 1.95(1.23–3.09) |
|  | rs9939609TA+AA | 395 (53%) | 350 (63%) | 7.27 | 0.007 | 1.50(1.12–2.00) |
|  | rs9939609T | 1030 (67%) | 640 (60%) | – | – | Reference |
|  | rs9939609A | 470 (31%) | 440 (40%) | 9.44 | 0.002 | 1.39(1.13–1.72) |
|  |  |  |  |  |  |  |
| rs9926289G/A | rs9926289GG | 305 (41%) | 176 (32%) | – | – | Reference |
|  | rs9926289GA | 348 (46%) | 279 (51%) | 3.88 | 0.049 | 1.37(1.00–1.88) |
|  | rs9926289AA | 97 (13%) | 95 (17%) | 4.85 | 0.028 | 1.65(1.06–2.57) |
|  | rs9926289GA+AA | 445 (59%) | 374 (68%) | 5.51 | 0.019 | 1.43(1.06–1.93) |
|  | rs9926289G | 958 (64%) | 631 (57%) | – | – | Reference |
|  | rs9926289A | 542 (36%) | 469 (43%) | 5.87 | 0.015 | 1.29(1.05–1.59) |
|  |  |  |  |  |  |  |
| rs9930506A/G | rs9930506AA | 283 (38%) | 165 (30%) | – | – | Reference |
|  | rs9930506AG | 326 (43%) | 267 (49%) | 1.24 | 0.264 | 1.20(0.87–1.65) |
|  | rs9930506GG | 141 (19%) | 118 (21%) | 3.52 | 0.061 | 1.48(1.00–2.24) |
|  | rs9930506AG+GG | 467 (62%) | 385 (70%) | 2.50 | 0.114 | 1.27(0.94–1.72) |
|  | rs9930506A | 892 (59%) | 597 (54%) | – | – | Reference |
|  | rs9930506G | 608 (41%) | 503 (46%) | 3.72 | 0.054 | 1.22(1.00–1.50) |
|  |  |  |  |  |  |  |
| rs9932754C/T | rs9932754CC | 183 (25%) | 106 (19%) | – | – | Reference |
|  | rs9932754TC | 332 (44%) | 260 (47%) | 4.00 | 0.046 | 1.48(1.01–2.10) |
|  | rs9932754TT | 235 (31%) | 184 (34%) | 2.66 | 0.103 | 1.40(0.93–1.94) |
|  | rs9932754TC+TT | 567 (75%) | 444 (81%) | 4.03 | 0.045 | 1.44(1.01–2.07) |
|  | rs9932754C | 698 (47%) | 472 (43%) | – | – | Reference |
|  | rs9932754T | 802 (53%) | 628 (57%) | 2.08 | 0.150 | 1.16(0.95–1.42) |
|  |  |  |  |  |  |  |
| ***GNB3*** |  |  |  |  |  |  |
|  |  |  |  |  |  |  |
| rs1129649T/C | rs1129649TT | 357 (48%) | 172 (31%) | – | – | Reference |
|  | rs1129649TC | 319 (42%) | 315 (57%) | 24.97 | 5.81E-07 | 2.20(1.62–3.00) |
|  | rs1129649CC | 74 (10%) | 63 (12%) | 8.99 | 2.71E-03 | 2.28(1.33–3.91) |
|  | rs1129649TC+CC | 393 (52%) | 378 (69%) | 26.79 | 2.27E-07 | 2.21(1.64–2.99) |
|  | rs1129649T | 1033 (69%) | 659 (60%) | – | – | Reference |
|  | rs1129649C | 467 (31%) | 441 (40%) | 19.76 | 8.76E-06 | 1.63(1.32–2.03) |
|  |  |  |  |  |  |  |
| rs5443C/T | rs5443CC | 396 (57%) | 207 (38%) | – | – | Reference |
|  | rs5443CT | 312 (36%) | 261 (47%) | 14.25 | 1.60E-04 | 1.82(1.33–2.48) |
|  | rs5443TT | 42 (07%) | 82 (15%) | 35.62 | 2.39E-09 | 5.45(3.12–9.51) |
|  | rs5443CT+TT | 354 (43%) | 343 (62%) | 25.37 | 4.73E-07 | 2.15(1.60–2.90) |
|  | rs5443C | 1104 (74%) | 675 (61%) | – | – | Reference |
|  | rs5443T | 396 (26%) | 425 (39%) | 37.44 | 9.45E-10 | 2.00(1.60–2.50) |
|  |  |  |  |  |  |  |

n; represents the number of subjects; χ^2^, Chi-square value; OR, Odds Ratio.
